# Reflecting Emotional Intelligence: How Mindsets Navigate Academic Engagement and Burnout Among College Students

**DOI:** 10.3390/bs15091261

**Published:** 2025-09-15

**Authors:** Yunshan Jiang, Jianwei Zhang, Wenfeng Zheng, Guangxia Guo, Wenya Yang

**Affiliations:** 1 School of Education, Beijing Institute of Technology, Beijing 102488, China; jiangyunshann@163.com (Y.J.); guoguangxia1997@163.com (G.G.); yangwenyah@yeah.net (W.Y.); 2 Faculty of Education, Shaanxi Xueqian Normal University, Xi’an 710100, China; zwf19_94@163.com

**Keywords:** EI mindsets, academic engagement, academic burnout, regulatory focus, performance-prove goal orientation

## Abstract

Despite the growing recognition of emotional intelligence (EI) and its significant associations with academic outcomes, less is known about the underlying mechanisms through which EI mindsets affect academic engagement and burnout. Drawing on regulatory focus theory and social comparison theory, this study aims to reveal how different types of EI mindsets influence college students’ academic engagement and burnout through regulatory focus (i.e., promotion and prevention focus) and further examines the moderating role of performance-prove goal orientation—defined as the motivation to demonstrate competence and outperform others—in these pathways. To test these associations, we conducted two studies. A scenario experiment (Study 1) indicates that a growth mindset of EI (GMOE) has the potential to enhance academic engagement while reducing academic burnout, whereas a fixed mindset of EI (FMOE) exhibits the opposite pattern. Study 2, based on three-wave data, demonstrates that GMOE is positively associated with academic engagement and negatively associated with academic burnout via promotion focus, whereas FMOE is positively associated with academic burnout and negatively associated with academic engagement through prevention focus. Of note, performance-prove goal orientation moderates these pathways: Individuals with higher levels of performance-prove goal orientation exhibit a weakened indirect effect of GMOE on academic engagement via promotion focus, whereas those with lower levels of performance-prove goal orientation display a strengthened version of this pathway. Conversely, the indirect effect of FMOE on academic burnout through prevention focus is stronger when performance-prove goal orientation is high and weaker when it is low. Theoretical and practical implications are also discussed.

## 1. Introduction

In spite of significant advancements in understanding the connection between EI and academic performance (Quílez-Robres et al., 2023; Ubago-Jimenez et al., 2024), current research on the nature of EI remains controversial, with two diverging perspectives: the ability view (a malleable capacity to recognize, understand, regulate, and utilize emotions; Brackett et al., 2006; Goleman, 1995; Salovey & Sluyter, 1997) and the trait view (a stable personality trait related to emotion processing; Petrides et al., 2007; Sánchez-Ruiz et al., 2010). As noted above, these perspectives, to some extent, suggest that people may differ in their beliefs about the nature of EI. EI mindsets, also known as implicit theories of EI, refer to individuals’ beliefs about the malleability of EI—specifically, whether it can be developed or remains fixed.

People hold specific mindsets or implicit beliefs that encompass diverse mental representations of the nature and workings of the self, their relationships, and the world (Dweck, 2008). Although a substantial body of research has demonstrated the significant impact of mindsets across various domains—such as intelligence (Burnette et al., 2023; Dweck & Yeager, 2019), personality (Dweck, 2008; Yeager et al., 2013), emotion (Kneeland & Kisley, 2023; Tamir et al., 2007), and interpersonal relationships (Knee et al., 2004)—scholarly attention has only recently turned to their unique implications in the domain of EI. While a few studies have begun to explore EI mindsets and their associations with academic outcomes, the underlying mechanisms through which such mindsets exert influence remain unclear (Costa & Faria, 2020, 2025). Moreover, existing research has largely focused on secondary school students and overt academic outcomes (e.g., GPA), leaving the question of whether and how EI mindsets shape college students’ academic engagement and burnout largely unexplored.

In higher education, academic engagement and burnout not only reflect students’ current academic performance but also exert long-term effects on their learning motivation, psychological development, and career trajectories (Meng & Zhang, 2023; Reyes-de-Cózar et al., 2023). Academic engagement reflects students’ positive psychological state toward learning, referring to the constructive attitudes and behaviors they exhibit during the learning process, such as concentration, effort, initiative, and emotional investment (Reeve & Tseng, 2011). In contrast, academic burnout reflects students’ negative psychological state, referring to the adverse attitudes and behaviors arising from academic pressure or a lack of interest in learning (Lian et al., 2005; Maslach & Jackson, 1981). Prior research suggests that negative academic states such as burnout can hinder classroom learning and pose risks to students’ long-term personal development (Reyes-de-Cózar et al., 2023). Therefore, systematically examining the impact of EI mindsets on college students’ academic engagement and burnout is essential—not only to expand the scope of implicit theories but also to inform strategies for improving students’ academic and psychological adjustments.

How do EI mindsets influence academic engagement and burnout? A large body of research has argued that domain-specific mindsets—core beliefs that orient people to a particular set of expectations, attributions, and goals—alter not only how individuals interpret their experiences but also their motivational orientations and academic outcomes (Blackwell et al., 2007; Dweck & Yeager, 2019). Accordingly, EI mindsets, as a domain-specific mindset within the field of emotional intelligence, are likely to influence students’ academic engagement and burnout by shaping their motivational tendencies. To this end, we draw on regulatory focus theory (Higgins, 1997) and develop a theoretical model for understanding the effects of EI mindsets on academic engagement and burnout. This theory examines the relationship between individuals’ motivation and the way in which they engage in achieving their goals, suggesting that individuals generally adopt either a prevention focus (i.e., avoiding failure) or a promotion focus (i.e., pursuing success) to fulfill achievement-related tasks (Higgins, 2000). Specifically, we suggest that GMOE—the implicit belief that EI is malleable or can be developed—may lead to promotion focus, thereby enhancing positive outcomes (e.g., learning engagement; H. Liu et al., 2020). In contrast, we argue that FMOE—the implicit belief that EI is fixed or unchangeable—may lead to prevention focus, thereby leading to negative outcomes (e.g., school burnout; Yasuda & Goegan, 2023). Taken together, the present research integrates competing perspectives to indicate that EI mindsets may have both positive and negative effects on college students’ academic engagement and burnout by identifying regulatory focus as a theoretically relevant mediator.

**Figure 1:**
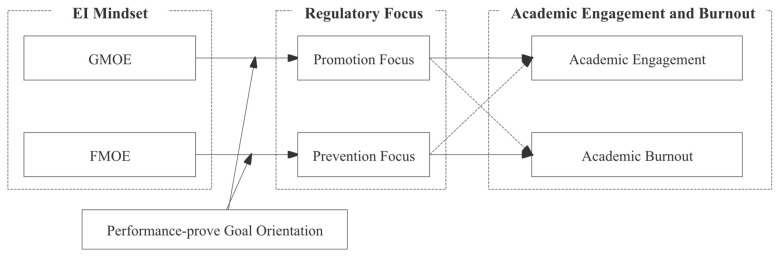
Research framework.

Furthermore, EI mindsets are unlikely to be separated from the social comparison context. Social comparison is a ubiquitous process that impacts almost every domain of human life, affecting individuals’ emotions, perceptions, and attitudes (Strickhouser & Zell, 2015). Social comparison theory (Festinger, 1954) suggests that certain dispositional characteristics may influence how an individual interprets relevant information in a social comparison environment. Notably, dispositional goal orientations help explain why individuals engage in achievement behaviors (Sung et al., 2024). For example, particularly when confronting emotional events, such as upward comparison, peer conflict, and academic failure in achievement-related environments, performance-prove goal orientation reflects individuals’ tendency to show competence and surpass others (Elliot & Church, 1997). Accordingly, we propose that the effect of EI mindsets differs across college students, depending on their level of performance-prove goal orientation—those with higher levels are more likely to interpret the malleability of EI less favorably. In this respect, the current study identifies performance-prove goal orientation as a potential boundary condition for the effects of EI mindsets. High-performance-prove goal orientation individuals tend to perceive others’ performance as self-evaluative threats rather than instructive role models (Downes et al., 2021), thereby hindering their learning and growth. Therefore, we expect that performance-prove goal orientation might diminish the positive downstream consequences of EI mindsets. Figure 1 depicts our theoretical model.

Our research offers several contributions to theory and practice. First, we extend literature on EI mindsets. Although the importance of EI mindsets has received growing attention in literature, little research has examined why they influence academic engagement and burnout. To address this gap, we employ a scenario-based experimental method to identify the potential causal relationship between EI mindsets and academic engagement and burnout. Second, we clarify the underlying mechanisms by integrating regulatory focus theory and social comparison theory. Previous research has not systematically explored the psychological processes through which EI mindsets affect academic outcomes. Our study responds to theoretical calls for the elaboration of mechanisms and the identification of boundary conditions by testing the mediating roles of promotion and prevention focus and identifying the moderating effect of performance-prove goal orientation (Cabello & Fernández-Berrocal, 2015; Costa & Faria, 2022). Finally, our findings offer important practical implications for higher education. Interventions might aim to cultivate students’ GMOE in order to enhance academic engagement and reduce burnout. Moreover, educators could pay close attention to students’ regulatory focus tendencies and levels of performance-prove goal orientation, providing tailored support accordingly.

## 2. Theoretical Background and Research Hypothesis

People’s implicit beliefs about the malleability of personal attributes (ranging from intelligence to body weight) shape the way they understand, experience, and respond to the world. These mindsets range from believing that traits are malleable (i.e., growth mindset) to believing that they are immutable (i.e., fixed mindset; Dweck, 1986). Most early work focused on mindsets related to intelligence, but implicit theories can now be applied to understand a range of traits, abilities, and outcomes (Zedelius et al., 2017). Mindsets in specific domains have been shown to profoundly affect various aspects of functioning, such as motivation, mental health, and higher-level performance (Hoyt et al., 2023; Job et al., 2015). The expansion of mindsets across various domains demonstrates their universality and predictive power. Although the growth mindset theory has long received extensive empirical support, recent research has begun to re-examine the applicability and underlying mechanisms of its intervention effects.

In particular, a major meta-analysis by Macnamara and Burgoyne (2023) indicated that the overall impact of growth mindset interventions on academic achievement is small and may have been overstated due to methodological shortcomings, reporting inconsistencies, and publication bias. In contrast, a meta-analysis by Burnette et al. (2023) reported more positive findings, emphasizing that interventions tend to be more effective when targeting specific focal groups (e.g., underserved or low-achieving students) and when delivered with high implementation fidelity and motivational alignment. Moreover, Sisk et al. (2018) pointed out that growth mindset interventions exhibit considerable heterogeneity in effect sizes, which—together with their overall limited impact—has raised questions regarding their generalizability and practical utility. Notably, Yeager and Dweck (2020) also acknowledged this issue, suggesting that future research should develop more nuanced and context-sensitive theoretical models to identify moderators and boundary conditions. Taken together, while implicit theories continue to offer valuable insights in educational psychology, their limitations call for a more cautious interpretation and application of the theory. This ongoing academic debate further underscores the importance of clarifying the mechanisms and contextual boundaries of domain-specific mindsets (e.g., EI mindsets).

In light of the growing controversies surrounding implicit theories, it is particularly important to further clarify their applicability within specific domains. Among them, EI mindsets, as an emerging and underexplored form of implicit theory, merit closer scholarly attention. Although prior research has extended the concept of mindsets to the emotion-related domain (De France & Hollenstein, 2021; Moumne et al., 2021; Tamir et al., 2007), systematic attention to mindsets specifically related to EI is still limited. Unlike Dweck’s notion of the intelligence mindset—which refers to individuals’ beliefs about whether intellectual ability can be improved through effort (Dweck & Leggett, 1988; Dweck, 1999)—an EI mindset specifically refers to individuals’ beliefs about whether they can enhance their ability to recognize, understand, express, and regulate emotions and to use emotions to facilitate thinking through learning and practice. Conceptually, EI mindsets are also clearly distinct from emotion mindsets. The former emphasizes beliefs about the malleability of EI, whereas the latter more broadly concerns whether individuals believe that their emotional or affective states are changeable (Costa & Faria, 2025). Moreover, empirical studies have demonstrated that implicit theories are highly domain-specific, meaning that individuals’ beliefs about malleability in one domain do not necessarily transfer to another (King & dela Rosa, 2019). Thus, applying insights from intelligence or emotion mindsets to EI mindsets may be conceptually and empirically unjustified, reinforcing the need for targeted investigation in this domain.

Accordingly, the present study aims to further extend the framework of implicit theories of EI to facilitate a more comprehensive understanding of EI mindsets and their associations with academic engagement and burnout. Drawing on implicit theories and regulatory focus theory (Dweck, 1986; Higgins, 1997), we propose that individuals with a GMOE tend to view EI as malleable and improvable. This belief leads them to adopt an advancement-oriented outlook, thereby orienting them toward a promotion focus (Burnette et al., 2023). Such a motivational orientation makes them more likely to construe EI-related academic tasks as opportunities for growth and progress, which in turn stimulates proactive strategies (e.g., striving for self-actualization and continuous improvement) and ultimately enhances academic engagement (Yadav & Dhar, 2021). Moreover, these individuals tend to view EI-related academic demands positively, interpreting the resulting stress as a challenge, thereby reducing the risk of academic burnout (Yasuda & Goegan, 2023). In contrast, individuals with an FMOE tend to perceive EI as fixed and difficult to improve. This belief leads them to adopt an avoidance-oriented perspective, thereby orienting them toward a prevention focus (B. Liu et al., 2024). Such a motivational orientation causes them to interpret EI-related academic tasks as threats or evaluative situations, which in turn drives them to adopt avoidance-oriented strategies (e.g., striving to avoid failure or negative evaluation), thereby undermining academic engagement (Deng et al., 2022). At the same time, the persistent tension and resource depletion associated with this orientation further increase the likelihood of academic burnout (Oertig et al., 2013).

In addition, drawing on social comparison theory (Festinger, 1954), upward social comparisons typically highlight an individual’s perceived deficiencies relative to others, thereby triggering negative reactions such as anxiety and self-doubt. Individuals with high levels of performance-prove goal orientation are more inclined to engage in upward comparisons, which may either undermine the promotion focus elicited by GMOE or exacerbate the prevention focus triggered by FMOE (Downes et al., 2021; Muller & Fayant, 2010). Building on these theoretical perspectives, the present study seeks to elucidate how and under what conditions EI mindsets contribute to distinct patterns of academic engagement and burnout among college students.

### 2.1. Linking EI Mindsets with Academic Engagement and Burnout

In college life, students’ academic engagement and academic burnout represent two distinct psychological states. Academic engagement typically refers to individuals’ active, focused, and sustained involvement in learning activities, which is closely associated with academic achievement and is also predictive of positive psychological adjustment (Martínez et al., 2019; Y. Zhang et al., 2018). In contrast, academic burnout is characterized by emotional exhaustion, a cynical or detached attitude toward learning, and diminished academic efficacy and has been closely linked to reduced academic functioning, as well as psychological problems, such as anxiety and depression (Olson et al., 2025; Xia et al., 2025).

Existing research has shown that individuals’ mindsets are closely associated with key academic outcomes such as academic engagement and academic burnout (Vestad & Bru, 2024; J. Zhang et al., 2025). Meanwhile, although a substantial body of research has demonstrated that EI significantly predicts students’ academic adaptation and achievement (MacCann et al., 2020; Quílez-Robres et al., 2023), the specific mechanisms through which EI mindsets influence students’ academic engagement and burnout remain underexplored. Within the framework of implicit theories of EI, students who hold GMOE tend to believe that EI can be improved through learning and effort. This belief may guide them to approach EI-related academic tasks with an orientation toward growth and progress and to adopt proactive coping strategies, which may in turn be reflected in higher levels of academic engagement (Taghani & Razavi, 2022). Moreover, as these students are inclined to perceive academic demands as opportunities for the development and potential enhancement of EI, they are more likely to actively mobilize resources for self-regulation in pursuit of growth and potential gains, thereby facing a relatively lower risk of academic burnout (Izadpanah, 2023). By contrast, students who hold FMOE typically conceive of EI as an inborn and unalterable attribute. This assumption may lead them to interpret EI-related academic tasks as evaluative threats that risk exposing shortcomings in their EI, thereby fostering a tendency to adopt avoidance-based coping strategies, which in turn can manifest in reduced academic engagement (Zhong et al., 2023). Furthermore, because they often devote their energy to masking perceived deficits in EI rather than fostering its growth and development, this maladaptive tendency may place them at a heightened risk of academic burnout (Vizoso et al., 2019). We therefore hypothesize the following:

**H1a.**  *GMOE is positively related to (1) academic engagement and negatively related to (2) academic burnout.*

**H1b.**  *FMOE is positively related to (1) academic burnout and negatively related to (2) academic engagement.*

### 2.2. The Mediating Role of Regulatory Focus

Regulatory focus refers to the way individuals regulate their motivational, cognitive, and behavioral processes during goal pursuit and consists of two types: promotion focus and prevention focus (Higgins, 1997). Individuals with a promotion focus prioritize growth and progress and tend to adopt proactive strategies that emphasize the enhancement of personal capabilities; in contrast, those with a prevention focus prioritize avoiding failure, often employing defensive strategies aimed at fulfilling obligations. Existing research has revealed a hierarchical association between mindsets and regulatory focus, indicating that a growth mindset can foster positive development through a promotion focus, whereas a fixed mindset may lead to conservative coping strategies through a prevention focus (Sue-Chan et al., 2012).

From the perspective of implicit theories of EI, different EI mindsets may activate distinct motivational orientations. Specifically, students who hold GMOE regard EI as malleable, which makes them more likely to interpret EI-related academic tasks as opportunities for improvement and to exhibit a constructive, improvement-oriented disposition. This motivational orientation aligns with a promotion focus that emphasizes growth and advancement (Higgins, 1998; Kümmel & Kimmerle, 2020). In contrast, students who hold FMOE view EI as stable and unchangeable, making them more likely to construe EI-related academic contexts as threats and to display a tendency oriented toward risk avoidance. This motivational orientation is consistent with a prevention focus that prioritizes security and the avoidance of negative outcomes (Lockwood et al., 2002; Zarrinabadi & Saberi Dehkordi, 2024).

Meanwhile, regulatory focus, as a motivational orientation, influences how students construe academic demands and make subsequent coping choices, thereby affecting both academic engagement and burnout. Specifically, individuals with a promotion focus are guided by aspirations and growth-oriented goals, making them more likely to attend to potential gains and achievements (Hoch et al., 2024). Empirical studies have shown that this orientation enhances academic engagement by strengthening self-efficacy, eliciting positive emotions, and improving learning motivation and self-regulation (Deng et al., 2022; Lanaj et al., 2012; H. Liu et al., 2020). In addition, a promotion focus encourages individuals to perceive academic requirements as developmental challenges rather than threats, which buffers resource depletion under academic stress and thereby reduces the risk of academic burnout (Kwon, 2022; Yasuda & Goegan, 2023).

In contrast, individuals with a prevention focus are highly sensitive to potential losses and primarily driven by security needs, which keeps them in a state of heightened vigilance when engaging in academic tasks (Higgins, 1997). Although such vigilance may be adaptive for risk avoidance, it often depletes self-regulatory resources (Crowe & Higgins, 1997) and is accompanied by tension, anxiety, and feelings of helplessness (Brockner & Higgins, 2001). Over time, this orientation undermines persistence and increases tendencies toward withdrawal, thereby elevating the likelihood of academic burnout (Oertig et al., 2013). Furthermore, prevention focus may also diminish students’ learning engagement by reducing self-efficacy and eliciting negative emotional experiences (Deng et al., 2022; H. Liu et al., 2020). Therefore, this study proposes that individuals’ EI mindsets shape their academic outcomes through regulatory focus: those with GMOE are inclined to adopt a promotion focus, which fosters academic engagement while alleviating burnout, whereas those with FMOE tend to adopt a prevention focus, which diminishes engagement and heightens the risk of burnout. We therefore hypothesize the following:

**H2a.**  *Promotion focus is positively related to (1) academic engagement and negatively related to (2) academic burnout.*

**H2b.**  *Prevention focus is positively related to (1) academic burnout and negatively related to (2) academic engagement.*

**H3a.**  *GMOE has a positive indirect effect on (1) academic engagement and a negative indirect effect on (2) academic burnout, mediated by promotion focus.*

**H3b.**  *FMOE has a positive indirect effect on (1) academic burnout and a negative indirect effect on (2) academic engagement, mediated by prevention focus.*

### 2.3. The Moderating Role of Performance-Prove Goal Orientation

Social comparison theory posits that when evaluative standards are ambiguous, individuals tend to compare themselves with others to form relative judgments about their abilities and opinions (Festinger, 1954). In college settings, academic evaluation criteria are often vague and context-dependent, thereby compelling students to engage in frequent social comparisons to demonstrate their competence and gain recognition from peers and instructors (Lee, 2022). Within this process, performance-prove goal orientation—a motivational disposition characterized by the desire to validate one’s abilities and receive positive evaluations from others—is strongly aligned with social comparison mechanisms (VandeWalle, 1997). Students with this orientation are particularly inclined to engage in upward social comparisons, as social comparison is considered an inherent component of performance-prove goal orientation (Dietz et al., 2015).

Existing research has shown that performance goals are closely related to individuals’ implicit beliefs about plasticity (Dweck, 1986; Dweck & Leggett, 1988). Therefore, performance-prove goal orientation may serve as a critical boundary condition influencing the relationship between college students’ EI mindsets and regulatory focus. Specifically, students with high performance-prove goal orientation tend to compare their achievements with others to ensure their superiority, thereby focusing more on external evaluations in academic and social interactions and emphasizing “outperforming others” rather than actual growth (Darnon et al., 2010). Additionally, individuals with high performance-prove goal orientation often process social information through evaluative thinking and are more likely to view abilities as fixed traits (Watson et al., 2013). Students with GMOE are more likely to develop a promotion focus. However, when these students simultaneously exhibit a strong performance-prove goal orientation, their excessive focus on external outcomes, such as “how to be better than others”, may undermine their intrinsic growth motivation, thereby suppressing the development of a promotion focus. Accordingly, we propose the following hypothesis:

**H4a.**  *Performance-prove goal orientation negatively moderates the relationship between GMOE and promotion focus.*

Based on Hypotheses 3a and 4a, we further propose the following:

**H5a.**  *Performance-prove goal orientation negatively moderates the mediating effect of promotion focus in the relationship between GMOE and academic engagement.*

Students with FMOE are more likely to develop a prevention focus. When these students simultaneously exhibit a high performance-prove goal orientation, they are inclined to engage in upward social comparison, intensifying their fear of failure (Giel et al., 2020). In this scenario, students may become more preoccupied with potential negative evaluations of their EI and academic performance by others. This “upward comparison anxiety” reinforces their motivation to avoid failure and evade challenges, thereby strengthening the prevention focus. Based on this, we propose the following hypothesis:

**H4b.**  *Performance-prove goal orientation positively moderates the relationship between FMOE and prevention focus.*

Based on Hypotheses 3b and 4b, we further propose the following:

**H5b.**  *Performance-prove goal orientation positively moderates the mediating effect of prevention focus in the relationship between FMOE and academic burnout.*

## 3. Study 1

### 3.1. Samples and Procedure

A total of 205 undergraduates from a Chinese college participated in the study (57.1% male; *M_age* = 19.46, *SD* = 2.08). All participants voluntarily took part in the experiment and provided informed consent. Participants were randomly assigned to either the GMOE condition (*n* = 102) or the FMOE condition (*n* = 103) using a computer-generated randomization procedure. The study employed a 2 (group: growth vs. fixed) × 2 (time: pre-test vs. post-test) mixed factorial design, with group as the between-subjects factor and time as the within-subjects factor.

The procedure comprised three stages. First, participants completed a baseline assessment of their EI mindsets. Next, those in the GMOE condition read an article emphasizing that “EI can be developed,” whereas participants in the FMOE condition read an article emphasizing that “EI is innate and difficult to change.” These materials were used to manipulate participants’ EI mindsets. Following the manipulation, participants completed a second set of questionnaires assessing their EI mindsets, academic engagement, and academic burnout.

### 3.2. Measures

EI Mindset. Based on Dweck’s (1999) implicit theories framework, we developed and adapted a scale to measure EI mindset. In constructing the scale, we drew on existing instruments designed to assess implicit theories of EI (e.g., Costa & Faria, 2023). To ensure semantic clarity and contextual appropriateness, the items were further revised to account for Chinese students’ linguistic habits, cultural norms, and educational context. The scale includes eight items: four items assessing GMOE (e.g., “I can fundamentally change my emotional intelligence”) and four items assessing FMOE (e.g., “My emotional intelligence is difficult for me to change”). Responses were given on a 6-point Likert scale (1 = completely disagree, and 6 = completely agree). The scale showed high internal consistency in this study (Cronbach’s *α* = 0.858 for GMOE and 0.824 for FMOE).

Academic engagement. Academic engagement was measured using the 22-item scale developed by Reeve and Tseng (2011). A sample item is “I enjoy learning new things in class.” Items were rated on a 5-point Likert scale (1 = strongly disagree, 5 = strongly agree). The scale showed high reliability (Cronbach’s *α* = 0.902).

Academic burnout. Academic burnout was measured using the 12-item scale developed by Maslach and Jackson (1981), which was adapted and validated for Chinese college students by Lian et al. (2005). A sample item is “I am always bored with studying.” Responses were rated on a 5-point Likert scale (1 = strongly disagree, 5 = strongly agree). The scale exhibited good internal consistency (Cronbach’s *α* = 0.805).

### 3.3. Results

#### 3.3.1. Manipulation Checks

Prior to the experimental manipulation, participants’ EI mindsets were assessed to establish baseline equivalence. After the manipulation, the same scale was administered again. Paired-samples *t*-tests revealed that, within the targeted mindset dimensions, scores significantly increased within groups. In the growth mindset group, post-test scores on GMOE (*M* = 4.41, *SD* = 0.99) were significantly higher than pre-test scores (*M* = 3.34, *SD* = 0.74) (*t*(101) = 10.39, *p* < 0.001). In the fixed mindset group, post-test scores on FMOE (*M* = 4.82, *SD* = 0.84) were significantly higher than pre-test scores (*M* = 3.17, *SD* = 0.91) (*t*(102) = 13.88, *p* < 0.001).

Independent-samples t-tests further confirmed the effectiveness of the manipulation. After the manipulation, the growth mindset group scored significantly higher on GMOE than the FMOE group (*M* = 4.41 vs. 2.18) (*t*(203) = 14.96, *p* < 0.001). Conversely, the FMOE group scored significantly higher on FMOE than the GMOE group (*M* = 4.82 vs. 2.12) (*t(199.89)* = 21.56, *p* < 0.001). Baseline scores did not significantly differ between groups in either GMOE (*M* = 3.34 vs. 3.26; *t*(203) = 0.64, *p* = 0.521) or FMOE (*M* = 3.25 vs. 3.17; *t*(203) = 0.70, *p* = 0.486), indicating successful randomization. Together, these results support the validity and effectiveness of the EI mindset manipulation.

#### 3.3.2. Experimental Results

Independent-samples *t*-tests were conducted to examine the effects of the EI mindsets manipulation on academic engagement and academic burnout. Participants in the GMOE condition reported significantly higher levels of academic engagement (*M* = 4.16, *SD* = 0.45) than those in the FMOE condition (*M* = 2.76, *SD* = 0.52) (*t*(203) = 20.59, *p* < 0.001). In contrast, academic burnout was significantly lower in the GMOE group (*M* = 2.05, *SD* = 0.52) than in the FMOE group (*M* = 3.01, *SD* = 0.40) (*t*(190.23) = 14.82, *p* < 0.001). These findings suggest that GMOE facilitates academic engagement and buffers against burnout, whereas FMOE has the opposite effects.

### 3.4. Discussion

Although Study 1 employed a scenario-based experiment to demonstrate the causal impact of EI mindsets on academic engagement and burnout, it did not explore the underlying mechanisms. Given the conceptual overlap between EI and EI mindsets, an important concern is whether the observed effects of EI mindsets could be reduced to individual differences in EI. To address this, we conducted a supplementary analysis to test whether EI mindsets uniquely predict academic engagement and burnout beyond EI (see Appendix A)^1^. To further examine how and when EI mindsets influence academic engagement and burnout, we conducted a three-wave questionnaire survey.

## 4. Study 2

### 4.1. Samples and Procedure

Participants were undergraduate students recruited from a Chinese college through a three-wave questionnaire survey. To mitigate common method bias, data were collected at three time points, each spaced one week apart. At Time 1 (Week 1), 528 students completed measures of GMOE and FMOE, along with demographic information. At Time 2 (Week 3), they reported their levels of promotion focus, prevention focus, and performance-prove goal orientation. At Time 3 (Week 5), participants completed assessments of academic engagement and academic burnout.

To incentivize participation, students received a CNY 10 cash voucher upon full completion of the study. After removing incomplete, patterned, or invalid responses (e.g., blank items or straightlining), the final valid sample comprised 507 participants, yielding an effective response rate of 96.02%. Among them, 56.2% were male, and the average age was 20.93 years (*SD* = 3.52). A priori power analysis using G*Power 3.1.9 indicated that a sample size of 153 was sufficient to detect a medium effect size (*f*^2^ = 0.15) with 95% power at a 0.05 significance level, confirming that the final sample size was more than adequate.

### 4.2. Measures

EI Mindset. GMOE and FMOE were assessed using the revised EI mindset scale adapted from Dweck (1999), as in Study 1. Cronbach’s *α* was 0.903 for GMOE and 0.851 for FMOE.

Academic engagement. Academic engagement was measured using the 17-item scale developed by Reeve and Tseng (2011), as in Study 1. The scale showed excellent internal consistency (*α* = 0.960).

Academic burnout. Academic burnout was assessed using the 12-item scale developed by Maslach and Jackson (1981), also consistent with Study 1. Cronbach’s *α* for this scale was 0.944.

Promotion focus. Promotion focus was measured using a 6-item subscale from the regulatory focus scale developed by Zhao and Namasivayam (2012). A sample item is: “I often think about how to achieve my aspirations.” Items were rated on a 5-point Likert scale (1 = strongly disagree, 5 = strongly agree). The scale showed high reliability (*α* = 0.882).

Prevention focus. Prevention focus was assessed using the 8-item subscale from the same regulatory focus scale. A sample item is: “I often think about how to avoid failures in life.” Participants responded on a 5-point Likert scale. The scale demonstrated good internal consistency (*α* = 0.902).

Performance-prove goal orientation. Performance-prove goal orientation was measured using a 4-item scale developed by VandeWalle (1997), adapted for the Chinese college student population. A sample item is: “I care about letting others know that I am a good student.” Responses were made on a 6-point Likert scale (1 = completely disagree, and 6 = completely agree). The scale showed strong reliability (*α* = 0.916).

Control variables. We controlled for demographic variables (age, gender, grade, and discipline), as recommended by prior research. The results remained consistent, regardless of whether these variables were included.

### 4.3. Data Analysis

Data analyses were conducted in three stages. First, reliability, validity, descriptive statistics, correlation analyses, and common method bias tests were performed using SPSS 29.0. Second, confirmatory factor analysis (CFA) and structural equation modeling (SEM) were conducted using AMOS 26.0 to assess measurement model validity and test mediation paths, respectively. The significance of mediation effects was evaluated using the bias-corrected percentile bootstrap method. Third, hierarchical regression analyses and the PROCESS v4.0 macro were used to examine moderation and moderated mediation effects.

### 4.4. Results

#### 4.4.1. Common Method Bias Test

Considering the potential risk of common method bias arising from self-reported data, we adopted both procedural and statistical remedies. Procedurally, we emphasized anonymity, confidentiality, and the academic-only use of data and included several reverse-worded questionnaire items. Statistically, Harman’s single-factor test indicated that the first unrotated factor accounted for only 22.9% of the total variance, well below the commonly accepted threshold of 40%. Thus, common method bias was unlikely to have exerted a significant influence on the findings of this study.

#### 4.4.2. Descriptive Analysis

Table 1 presents the means, standard deviations, and correlation coefficients among variables. Results showed GMOE was positively correlated with promotion focus (*r* = 0.193, *p* < 0.01) and academic engagement (*r* = 0.205, *p* < 0.01) but negatively correlated with academic burnout (*r* = −0.165, *p* < 0.01). Conversely, FMOE was positively associated with prevention focus (*r* = 0.239, *p* < 0.01) and academic burnout (*r* = 0.191, *p* < 0.01) and negatively associated with academic engagement (*r* = −0.168, *p* < 0.01). These correlation results provide preliminary evidence for the relationships among the variables.

#### 4.4.3. Main and Mediating Effects

**Figure 2:**
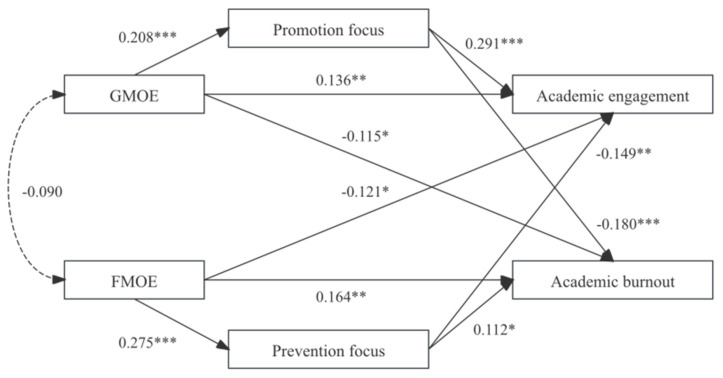
Standardized path coefficient diagram of the structural equation model. * *p* < 0.05, ** *p* < 0.01, *** *p* < 0.001.

Controlling for demographic variables (gender, grade, discipline, and age), we tested a structural equation model (SEM) with GMOE and FMOE as independent variables, academic engagement and academic burnout as dependent variables, and promotion and prevention focus as mediators. The model exhibited good fit indices: *χ*^2^/*df* = 1.27, RMSEA = 0.023, IFI = 0.977, TLI = 0.976, and CFI = 0.977. Path analysis results (see Figure 2) indicated the following significant effects: GMOE positively predicted academic engagement (*β* = 0.14, *SE* = 0.03, *p* < 0.05) and negatively predicted academic burnout (*β* = −0.12, *SE* = 0.03, *p* < 0.05), supporting Hypothesis 1a. FMOE positively predicted academic burnout (*β* = 0.16, *SE* = 0.04, *p* < 0.05) and negatively predicted academic engagement (*β* = −0.12, *SE* = 0.04, *p* < 0.05), supporting Hypothesis 1b. Promotion focus positively predicted academic engagement (*β* = 0.29, *SE* = 0.05, *p* < 0.05) and negatively predicted academic burnout (*β* = −0.18, *SE* = 0.05, *p* < 0.05), supporting Hypothesis 2a. Prevention focus positively predicted academic burnout (*β* = 0.11, *SE* = 0.05, *p* < 0.05) and negatively predicted academic engagement (*β* = −0.15, *SE* = 0.05, *p* < 0.05), supporting Hypothesis 2b.

To further examine mediating effects, we performed bias-corrected percentile bootstrap analyses (5000 resamples). As presented in Table 2, the 95% confidence intervals indicated significant mediation paths: Promotion focus significantly mediated between GMOE and academic engagement (indirect effect = 0.05, 95% CI = [0.02, 0.07]) and academic burnout (indirect effect = −0.03, 95% CI = [−0.05, −0.01]), supporting Hypothesis 3a. Prevention focus significantly mediated between FMOE and academic burnout (indirect effect = 0.02, 95% CI = [0.002, 0.05]) and academic engagement (indirect effect = −0.03, 95% CI = [−0.07, −0.01]), supporting Hypothesis 3b.

#### 4.4.4. Moderated Effects

To test whether performance-prove goal orientation moderates the relationships between EI mindsets and regulatory focus, GMOE, FMOE, and performance-prove goal orientation variables were mean-centered. Two interaction terms were then created: GMOE × performance-prove goal orientation and FMOE × performance-prove goal orientation. After controlling for relevant demographic variables, hierarchical linear regression analyses were performed (see Table 3). Results indicated that the interaction term GMOE × performance-prove goal orientation significantly and negatively predicted promotion focus (Model 3, *β* = −0.068, *p* < 0.05), suggesting that performance-prove goal orientation negatively moderates the relationship between GMOE and promotion focus. Additionally, the interaction term FMOE × performance-prove goal orientation significantly and positively predicted prevention focus (Model 6, *β* = 0.073, *p* < 0.01), indicating that performance-prove goal orientation positively moderates the relationship between FMOE and prevention focus.

**Figure 3:**
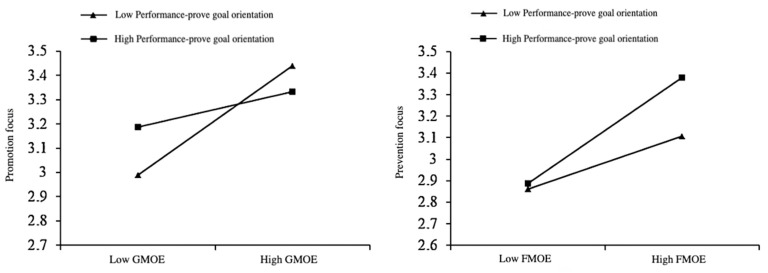
The moderating effect of performance-prove goal orientation.

To further illustrate the moderating role of performance-prove goal orientation, we plotted simple slopes at one standard deviation above and below the mean (see Figure 3). Results indicated that GMOE positively predicted promotion focus significantly for students with low performance-prove goal orientation (*β* = 0.226, *t* = 4.856, *p* < 0.001), whereas this effect was non-significant for students with high performance-prove goal orientation (*β* = 0.073, *t* = 1.609, *p* > 0.05). Thus, performance-prove goal orientation attenuated the positive relationship between GMOE and promotion focus, supporting Hypothesis 4a.

Conversely, FMOE positively predicted prevention focus significantly for students with low performance-prove goal orientation (*β* = 0.163, *t* = 4.137, *p* < 0.001), and this positive effect was strengthened for students with high performance-prove goal orientation (*β* = 0.328, *t* = 6.019, *p* < 0.001). Thus, performance-prove goal orientation enhanced the positive effect of FMOE on prevention focus, supporting Hypothesis 4b.

#### 4.4.5. Moderated Mediation Effects

We tested the moderated mediation model using the PROCESS v4.0 macro. As presented in Table 4, for the GMOE → promotion focus → academic engagement pathway, the indirect effect was significant at low levels of performance-prove goal orientation (effect = 0.064, Boot *SE* = 0.016, 95% CI [0.033, 0.096]). Additionally, the moderated mediation index was significant (index = −0.019, Boot *SE* = 0.009, 95% CI [−0.037, −0.003]), indicating that performance-prove goal orientation significantly moderated this mediation pathway. Thus, Hypothesis 5a was supported. For the FMOE → prevention focus → academic burnout pathway, the indirect effect was significant at high levels of performance-prove goal orientation (effect = 0.040, Boot *SE* = 0.018, 95% CI [0.007, 0.076]). The moderated mediation index was also significant (index = 0.009, Boot *SE* = 0.005, 95% CI [0.001, 0.020]), confirming that performance-prove goal orientation strengthened the mediation effect. Hence, Hypothesis 5b was supported.

### 4.5. Discussion

Although Study 1 demonstrated the causal impact of EI mindsets on academic engagement and burnout, Study 2 empirically tested and validated the full theoretical model and its hypotheses. The findings showed that EI mindsets robustly predict college students’ academic engagement, academic burnout, and regulatory focus, thereby corroborating and extending the experimental evidence from Study 1. Specifically, regulatory focus mediated the associations between mindsets of EI and academic engagement and burnout through two distinct pathways: promotion focus mediated the relationship between GMOE and increased academic engagement, as well as reduced academic burnout, whereas prevention focus mediated the relationship between FMOE and increased academic burnout and decreased academic engagement. Moderation analyses confirmed that performance-prove goal orientation attenuated the positive association between GMOE and promotion focus, thereby reducing its indirect impact on academic engagement, whereas it strengthened the relationship between FMOE and prevention focus, thereby increasing its indirect effect on academic burnout.

## 5. General Discussion

By integrating regulatory focus theory (Higgins, 1997) with social comparison theory (Festinger, 1954), we conducted two sequential empirical studies to systematically examine how and when EI mindsets influence college students’ academic engagement and burnout. We employed a scenario-based experimental design (Study 1) and provided the first causal evidence that EI mindsets influence academic engagement and burnout: GMOE significantly enhanced academic engagement and reduced burnout, whereas FMOE exhibited the opposite pattern. Building on this evidence, we further validated the full research model using a three-wave survey (Study 2). The results indicated that promotion focus partially mediated the positive effect of GMOE on increased academic engagement and reduced academic burnout, whereas prevention focus partially mediated the negative effect of FMOE on academic engagement and the positive effect on academic burnout. Moreover, performance-prove goal orientation served as a boundary condition, weakening the positive association between GMOE and promotion focus, thereby reducing its indirect effect on academic engagement while strengthening the positive association between FMOE and prevention focus, thereby increasing its indirect effect on academic burnout.

## 6. Theoretical Implications

This research enriches existing literature in three distinct ways. First, we contribute to research on EI mindsets by examining their relationships with academic engagement and burnout. To our knowledge, this research may be the first empirical investigation to comprehensively reveal the underlying mechanisms through which EI mindsets affect academic engagement and burnout. Given that research on EI mindsets remains in its nascent stage, only a handful of studies have tentatively explored their associations with academic outcomes (Costa & Faria, 2020, 2025). Through two complementary empirical studies—one of which employed a scenario-based experiment—we provide robust evidence for these effects and elucidate the underlying mechanisms. Our findings have significantly advanced the understanding of how EI mindsets differentially impact academic engagement and burnout, offering a novel and comprehensive framework to interpret their distinct associations.

Second, our work broadens the literature on regulatory focus by identifying its mediating role between EI mindsets and academic engagement and burnout. While a limited number of prior studies have explored the link between regulatory focus and domain-specific mindsets (e.g., coach’s mindset; Sue-Chan et al., 2012) and highlighted its importance for academic outcomes (Deng et al., 2022; Zhen et al., 2017), the influence of mindsets on regulatory focus has yet to be systematically validated within the EI domain. Moreover, the mediating role of regulatory focus in the relationship between EI mindsets and academic engagement and burnout remains largely unexplored. By identifying regulatory focus as a key mediating mechanism through which EI mindsets influence academic engagement and burnout, these findings deepen theoretical insights into regulatory focus within educational contexts and broaden its conceptual integration into the implicit theories framework.

Finally, we introduce the performance-prove goal orientation as a potential boundary condition in the relationship between EI mindsets and academic engagement and burnout, thereby contributing to research on both performance-prove goal orientation and social comparison theory. Previous studies have demonstrated that individuals’ mindsets are associated with their goal orientations (Burnette et al., 2013; Watson et al., 2013). For instance, a meta-analysis by Burnette et al. (2013) found a weak negative correlation between growth mindsets and performance-approach goals. However, the underlying mechanisms through which distinct EI mindsets shape individuals’ motivational tendencies, as well as academic engagement and burnout across varying levels of performance-prove goal orientation, remain largely underexplored. Our findings revealed that at higher levels of performance-prove goal orientation, GMOE’s beneficial effects on engagement via promotion focus diminished, while FMOE’s detrimental effects on burnout via prevention focus intensified. Therefore, the findings not only underscore the moderating role of performance-prove goal orientation in the pathway from EI mindsets to academic engagement and burnout, but also deepen our understanding of the boundary conditions under which social comparison processes shape motivational regulation.

## 7. Practical Implications

Our research carries a number of implications for higher education practice. First, we recommend that educators implement targeted interventions to enhance students’ GMOE. Our findings highlight that GMOE serves as a critical driver for fostering academic engagement and alleviating academic burnout among college students, thus meriting greater attention from educational practitioners. For example, teachers may incorporate GMOE-related content into mental health curricula to help students understand the malleability of EI and its connection to academic engagement and burnout. Meanwhile, research has shown that the provision of consistent and high-quality formative feedback by teachers can effectively enhance students’ growth mindsets (Murray et al., 2022) and thus may serve as a useful approach to fostering GMOE in everyday instruction. Additionally, school administrators may develop GMOE-themed lectures or workshops with interactive components to facilitate the internalization of the belief that EI can be developed through learning and practice. However, it is also important to recognize that the effectiveness of mindset interventions remains a topic of ongoing academic debate (Broda et al., 2018; Macnamara & Burgoyne, 2023; Paunesku et al., 2015; Sisk et al., 2018). In light of this, we suggest that future GMOE-focused interventions be implemented with high fidelity and contextual adaptation. To maximize effectiveness, such interventions should prioritize specific groups—such as students with academic difficulties or those with poor emotion regulation—and be embedded within supportive classroom or campus environments to optimize their impact (Burnette et al., 2023).

Second, given the critical role of regulatory focus in linking EI mindsets to academic engagement and burnout, we suggest that colleges emphasize cultivating students’ positive regulatory orientations, particularly promotion focus, when providing academic support. Prior research indicates that promotion focus is positively associated with self-efficacy, whereas prevention focus is not (Lanaj et al., 2012). Furthermore, academic skills training has been shown to effectively enhance students’ academic self-efficacy (Kordsalarzehi et al., 2025), making it a viable pathway to fostering students’ promotion focus. Specifically, colleges could integrate goal-setting, self-reflection activities, and targeted academic skills training, such as time management, task breakdown, and stress management, into curriculum design. These strategies collectively enhance students’ self-efficacy in learning contexts, thereby effectively facilitating and reinforcing their promotion orientation.

Finally, considering that performance-prove goal orientation may attenuate the beneficial effects of GMOE, it is of vital significance for educators to be highly sensitive to and attentive in situations that trigger competition, especially in group activities where students may feel pressured to outperform their peers. Accordingly, when designing instructional activities and assessment systems, educators should refrain from overemphasizing competitive rankings or publicly disclosing performance, thereby minimizing the likelihood of activating performance-prove goal orientation. For instance, instructors can implement evaluation criteria centered on individual progress, encourage students to reflect on the goals they have accomplished, and emphasize improvements relative to their previous performance rather than merely focusing on outcome-based rankings or grades. In doing so, this approach can enhance students’ promotion focus, thereby facilitating greater academic engagement and reducing academic burnout.

## 8. Limitations and Future Directions

Despite the valuable insights offered by our findings, several limitations should be acknowledged when generalizing the results of this research. First, all data in the present study were collected through self-report questionnaires, which may raise concerns about common method bias (Podsakoff et al., 2003). Although we employed a three-wave design to partially mitigate this issue, relying solely on single-source self-report data may still lead to inflated or biased associations among variables (Yao & Xu, 2024). Future research is therefore encouraged to adopt multi-method and multi-informant measurement strategies, such as combining self-report questionnaires with external assessments (e.g., peer ratings, teacher evaluations) and objective behavioral indicators (e.g., classroom participation, task completion), in order to improve the validity and explanatory power of model testing.

Second, this study has certain limitations in its selection of control variables, as it included only basic demographic variables (e.g., gender, age, and grade level) and did not account for individual-difference variables that may influence the model pathways (e.g., socioeconomic background, and personality traits). In addition, conceptually relevant constructs, such as intelligence mindsets or EI, were not included in the analysis, which may affect the accuracy of interpreting the conclusions. Future research is encouraged to incorporate a broader set of statistical control variables to enhance the robustness of model estimation and strengthen the theoretical validity of the findings.

Third, although Study 1 employed a scenario-based experiment to provide preliminary causal evidence for the effects of EI mindsets on academic engagement and burnout, the three-wave survey data used in Study 2 were essentially cross-sectional in nature. While this design offers certain temporal advantages, it remains insufficient to establish robust evidence for causal chains or directional pathways among the variables in the model, thereby limiting a deeper interpretation of the underlying mechanisms. Therefore, future research could employ longitudinal or experimental methods to systematically explore the dynamic mechanisms through which EI mindsets influence academic engagement and burnout, as well as to comprehensively evaluate the strength and boundary conditions of these effects.

Finally, this study focused on Chinese college students, and the use of a single cultural context may limit the external validity and generalizability across cultures of the findings. Previous research has shown that cultural norms (e.g., differing emotional display rules under individualistic vs. collectivistic orientations and socially acquired emotional expression norms) can shape how individuals express, perceive, and regulate emotions, thereby influencing their understanding of and responses related to EI (Ekermans, 2009). Therefore, future studies should not only include more culturally diverse samples but also conduct cross-cultural replications to examine whether the pathways through which EI mindsets influence academic engagement and burnout are culturally specific or cross-culturally stable.

## 9. Conclusions

This study examined how and under what conditions EI mindsets influence college students’ academic engagement and burnout. The results showed that GMOE enhanced students’ academic engagement and reduced academic burnout by strengthening promotion focus, whereas FMOE reduced academic engagement and intensified academic burnout through heightened prevention focus. In addition, performance-prove goal orientation emerged as a critical boundary condition, significantly moderating the first stage of these indirect pathways. Specifically, at higher levels of performance-prove goal orientation, the positive predictive effect of GMOE on promotion focus weakened, thereby reducing its indirect positive effect on academic engagement. Conversely, the positive predictive effect of FMOE on prevention focus strengthened, further amplifying its indirect negative effect on academic burnout. Based on the above findings, future research could further explore the diverse facets and underlying mechanisms through which EI mindsets affect academic engagement and burnout, thereby offering more targeted strategies to enhance college students’ academic engagement and reduce their risk of academic burnout.

## Figures and Tables

**Table 1:** Means, standard deviations, and correlations among variables.

Variable	*M*	*SD*	1	2	3	4	5	6	7	8	9	10	11
1. Gender (T1)	1.56	0.50	1										
2. Grade (T1)	2.73	1.15	0.033	1									
3. Discipline (T1)	2.42	1.04	0.304 **	−0.020	1								
4. Age (T1)	20.93	3.5	−0.019	0.351 **	0.050	1							
5. GMOE (T1)	3.13	0.98	0.007	−0.004	−0.024	−0.018	1						
6. FMOE (T1)	3.63	0.88	−0.052	−0.016	−0.033	−0.003	−0.072	1					
7. Promotion focus (T2)	3.26	0.75	−0.016	0.053	−0.028	0.003	0.193 **	−0.028	1				
8. Prevention focus (T2)	3.02	0.73	0.048	0.125 **	−0.020	0.068	−0.032	0.239 **	−0.114 *	1			
9. Performance-prove goal orientation (T2)	4.07	1.26	−0.028	0.020	−0.019	−0.028	−0.083	−0.088 *	−0.011	0.075	1		
10. Academic engagement (T3)	3.15	0.75	0.070	0.024	0.046	−0.013	0.205 **	−0.168 **	0.308 **	−0.205 **	−0.051	1	
11. Academic burnout (T3)	3.10	0.74	0.071	0.015	0.029	0.031	−0.165 **	0.191 **	−0.201 **	0.163 **	0.021	−0.134 **	1

Notes: * *p* < 0.05, ** *p* < 0.01.

**Table 2:** Bootstrap analysis of mediation effect significance.

Paths	Effect	Boot *SE*	Bias-Corrected 95%CI		
			Lower	Upper	*p*
GMOE → Promotion focus → Academic engagement	0.045	0.013	0.023	0.074	0.000
GMOE → Promotion focus → Academic burnout	−0.026	0.009	−0.049	−0.011	0.000
FMOE → Prevention focus → Academic burnout	0.023	0.013	0.002	0.054	0.035
FMOE → Prevention focus → Academic engagement	−0.033	0.013	−0.065	−0.012	0.002

**Table 3:** Regression analysis of moderating effects.

Variables	Promotion Focus			Prevention Focus		
	Model 1	Model 2	Model 3	Model 4	Model 5	Model 6
Gender	−0.018	−0.022	−0.010	0.083	0.104	0.086
Grade	0.038	0.038	0.031	0.071 *	0.071 *	0.070 *
Discipline	−0.016	−0.012	−0.03	−0.026	−0.022	−0.016
Age	−0.004	−0.003	−0.002	0.007	0.007	0.007
GMOE		0.147 ***	0.429 ***			
FMOE					0.211 ***	−0.056
Performance-prove goal orientation		0.002	0.224 *		0.057 *	−0.208 *
GMOE × performance-prove goal orientation			−0.068 *			
FMOE × performance-prove goal orientation						0.073 **
*R* ^2^	0.004	0.041	0.052	0.020	0.088	0.103
*F*	0.487	3.557 **	3.924 ***	2.501 *	8.067 ***	8.155 ***

* *p* < 0.05, ** *p* < 0.01, *** *p* < 0.001.

**Table 4:** Bootstrap test of moderated mediation effects.

Mediation Paths	Performance-Prove Goal Orientation	Effect	Boot SE	Boot LLCI	Boot ULCI
GMOE → Promotion focus →Academic engagement	*M* − *SD*	0.064	0.016	0.033	0.096
	M	0.040	0.011	0.019	0.062
	*M* + *SD*	0.021	0.013	−0.007	0.048
FMOE → Prevention focus → Academic burnout	*M* − *SD*	0.020	0.010	0.003	0.043
	*M*	0.031	0.014	0.005	0.060
	*M* + *SD*	0.040	0.018	0.007	0.076

## Data Availability

The raw data supporting the conclusions of this article will be made available by the authors upon request.

## Note

1 Based on a separate sample of 121 undergraduates (*M*age = 19.22; 62.81% male), we tested the incremental predictive validity of EI mindsets beyond EI. GMOE significantly improved the prediction of academic engagement (Δ*R*2 = 0.123, *p* < 0.001), and FMOE strengthened the capacity to predict academic burnout (Δ*R*2 = 0.045, *p* < 0.01). These findings support the incremental utility of EI mindsets beyond EI and demonstrate their distinct theoretical relevance for academic engagement and burnout. (See Appendix A for the full set of results.)

## Abbreviations

EI	emotional intelligence
GMOE	growth mindset of emotional intelligence
FMOE	fixed mindset of emotional intelligence

## Appendix A. Supplementary Analysis: Incremental Predictive Validity of EI Mindsets for Academic Engagement and Burnout Beyond EI (Full Version)

As a follow-up to Study 1, we conducted a supplementary analysis to examine whether EI mindsets predict academic engagement and burnout above and beyond EI. Although EI and EI mindsets share some conceptual overlap, they differ fundamentally in their theoretical connotations. This analysis aimed to test the incremental predictive validity of EI mindsets in forecasting college students’ academic engagement and burnout.

### Appendix A.1. Samples and Procedure

Participants were undergraduate students recruited from a Chinese college. A total of 138 students voluntarily participated and completed the survey, which included demographic information and measures of EI mindsets, EI, academic engagement, and academic burnout. After removing invalid responses (e.g., incomplete, patterned, or extreme answers), the final sample consisted of 121 valid participants, yielding an effective response rate of 87.5%. Among the final sample, 62.81% were male, and the average age was 19.22 years (*SD* = 1.11). A priori power analysis using G*Power 3.1.9 indicated that a minimum sample size of 119 would be sufficient to detect a medium effect size (*f*^2^ = 0.15) with 95% power at a significance level of α = 0.05, suggesting that the sample size was statistically adequate.

### Appendix A.2. Measures

EI Mindset. GMOE and FMOE were assessed using the revised EI mindset scale adapted from Dweck (1999), consistent with Study 1. The scale showed high internal consistency in this study (Cronbach’s α = 0.858 for GMOE, 0.824 for FMOE).

EI. EI was assessed using the 16-item scale developed by Law et al. (2004). A sample item is: “I have a good understanding of my own emotions.” Participants rated each item on a 7-point Likert scale (1 = strongly disagree, 7 = strongly agree). The scale demonstrated good internal consistency (Cronbach’s α = 0.832).

Academic engagement. Academic engagement was measured using the 22-item scale developed by Reeve and Tseng (2011), consistent with Study 1. A sample item is: “I enjoy learning new things in class.” Items were rated on a 5-point Likert scale (1 = strongly disagree, 5 = strongly agree). The scale showed high reliability (Cronbach’s α = 0.902).

Academic burnout. Academic burnout was measured using the 12-item scale developed by Maslach and Jackson (1981), consistent with Study 1. A sample item is: “I am always bored with studying.” Responses were recorded on a 5-point Likert scale (1 = strongly disagree, 5 = strongly agree). The scale exhibited good internal consistency (Cronbach’s α = 0.805).

Control variables. We controlled for demographic variables (age, gender, and discipline), as recommended by prior research. The results remained consistent, regardless of whether these variables were included.

### Appendix A.3. Results

Correlation analyses indicated that EI (*M* = 5.088, *SD* = 0.672) was significantly positively associated with GMOE (*M* = 3.508, *SD* = 0.891; *r* = 0.378, *p* < 0.001) and significantly negatively associated with FMOE (*M* = 3.372, *SD* = 0.901; *r* = −0.354, *p* < 0.001). These moderate correlations (Cohen, 2013) provide initial evidence of the association between EI and EI mindsets. Notably, the magnitude of these coefficients suggests that the EI mindset constitutes a distinct construct rather than a redundant measure of EI itself.

According to Hulland’s (1999) theoretical increment evaluation criteria, a new construct must demonstrate unique and incremental explanatory value beyond existing theoretical frameworks. In line with these criteria, we examined whether the new construct significantly improves the model’s explanatory power for the dependent variable (Δ*R*^2^), after controlling for relevant covariates.

As shown in Table A1, hierarchical regression analyses revealed that, after controlling for EI, GMOE added significant explanatory power to the academic engagement model (Δ*R*^2^ = 0.123, *F*(1, 115) = 21.175, *p* < 0.001), while FMOE significantly improved the explanatory power of the academic burnout model (Δ*R*^2^ = 0.045, *F*(1, 115) = 7.266, *p* < 0.01).

These results suggest that EI mindsets account for additional variance in academic engagement and burnout beyond EI itself, thereby satisfying the statistical criterion for theoretical increment (Hulland, 1999). This provides empirical support that EI mindsets represent a distinct and theoretically meaningful construct that contributes uniquely to the prediction of academic engagement and burnout.

**Table A1:** Hierarchical regression analysis results.

Variables	Academic Engagement			Academic Burnout		
	Model 1	Model 2	Model 3	Model 4	Model 5	Model 6
Gender	−0.002	0.064	0.038	0.044	−0.034	−0.050
Age	−0.058	−0.057	−0.061	0.050	0.049	0.049
Discipline	−0.065	−0.062	−0.054	0.080	0.077	0.074
EI		0.326 **	0.215 **		−0.384 **	−0.319 **
GMOE			0.218 **			
FMOE						0.140 **
*R* ^2^	0.032	0.211	0.333	0.038	0.248	0.292
*F*	1.296	7.740 ***	11.503 ***	1.536	9.541 ***	9.498 ***
△*R*^2^	0.032	0.179	0.123	0.038	0.210	0.045
△*F*	1.296	26.233 ***	21.175 ***	1.536	32.322 ***	7.266 **

Notes: ** *p* < 0.01, *** *p* < 0.001.

### Appendix A.4. Discussion

Study 1 employed a scenario-based experiment to examine the causal effects of EI mindsets on academic engagement and burnout. To further address potential conceptual overlap between EI and EI mindsets, we conducted a supplementary analysis testing the incremental predictive validity of EI mindsets beyond EI. The results provided additional empirical support for the unique contribution of EI mindsets in forecasting academic engagement and burnout.
